# Frameworks, Dimensions, Definitions of Aspects, and Assessment Methods for the Appraisal of Quality of Health Data for Secondary Use: Comprehensive Overview of Reviews

**DOI:** 10.2196/51560

**Published:** 2024-03-06

**Authors:** Jens Declerck, Dipak Kalra, Robert Vander Stichele, Pascal Coorevits

**Affiliations:** 1 Department of Public Health and Primary Care Unit of Medical Informatics and Statistics Ghent University Ghent Belgium; 2 The European Institute for Innovation through Health Data Ghent Belgium; 3 Faculty of Medicine and Health Sciences Heymans Institute of Pharmacology Ghent Belgium

**Keywords:** data quality, data quality dimensions, data quality assessment, secondary use, data quality framework, fit for purpose

## Abstract

**Background:**

Health care has not reached the full potential of the secondary use of health data because of—among other issues—concerns about the quality of the data being used. The shift toward digital health has led to an increase in the volume of health data. However, this increase in quantity has not been matched by a proportional improvement in the quality of health data.

**Objective:**

This review aims to offer a comprehensive overview of the existing frameworks for data quality dimensions and assessment methods for the secondary use of health data. In addition, it aims to consolidate the results into a unified framework.

**Methods:**

A review of reviews was conducted including reviews describing frameworks of data quality dimensions and their assessment methods, specifically from a secondary use perspective. Reviews were excluded if they were not related to the health care ecosystem, lacked relevant information related to our research objective, and were published in languages other than English.

**Results:**

A total of 22 reviews were included, comprising 22 frameworks, with 23 different terms for dimensions, and 62 definitions of dimensions. All dimensions were mapped toward the data quality framework of the European Institute for Innovation through Health Data. In total, 8 reviews mentioned 38 different assessment methods, pertaining to 31 definitions of the dimensions.

**Conclusions:**

The findings in this review revealed a lack of consensus in the literature regarding the terminology, definitions, and assessment methods for data quality dimensions. This creates ambiguity and difficulties in developing specific assessment methods. This study goes a step further by assigning all observed definitions to a consolidated framework of 9 data quality dimensions.

## Introduction

To face the multiple challenges within our health care system, the secondary use of health data holds multiple advantages: it could increase patient safety, provide insights into person-centered care, and foster innovation and clinical research. To maximize these benefits, the health care ecosystem is investing rapidly in primary sources, such as electronic health records (EHRs) and personalized health monitoring, as well as in secondary sources, such as health registries, health information systems, and digital health technologies, to effectively manage illnesses and health risks and improve health care outcomes [1]. These investments have led to large volumes of complex real-world data. However, health care is not obtaining the full potential of the secondary use of health data [2,3] because of—among other issues—concerns about the quality of the data being used [4,5]. Errors in the collection of health data are common. Studies have reported that at least half of EHR notes may contain an error leading to low-quality data [6-11]. The transition to digital health has produced more health data but not to the same extent as an increase in the quality of health data [12]. This will impede the potentially positive impact of digitalization on patient safety [13], patient care [14], decision-making [15], and clinical research [16].

The literature is replete with various definitions of data quality. One of the most used definitions for data quality comes from Juran et al [17], who defined data quality as “data that are fit for use in their intended operational, decision-making, planning, and strategic roles.” According to the International Organization for Standardization (ISO) definition, quality is “the capacity of an ensemble of intrinsic characteristics to satisfy requirements” (ISO 9000-2015). DAMA International (The Global Data Management Community: a leading international association involving both business and technical data management professionals) adapts this definition to a data context: “data quality is the degree to which the data dimensions meet requirements.” These definitions emphasize the subjectivity and context dependency of data quality [18]. Owing to this “fit for purpose” principle, the quality of data may be adequate when used for one specific task but not for another.

For example, when health data collected for primary use setting, such as blood pressure, are reused for different purposes, the adequacy of their quality can vary. For managing hypertension, the data’s accuracy and completeness may be considered adequate. However, if the same data are reused for research, for example, in a clinical trial evaluating the effectiveness of an antihypertensive, more precise and standardized measurements methods are needed. From the perspective of secondary use, data are of sufficient quality when they serve the needs of the specific goals of the reuser [4].

To ensure that the data are of high quality, they must meet some fundamental measurable characteristics (eg, data must be complete, correct, and up to date). These characteristics are called data quality dimensions, and several authors have attempted to formulate a complex multidimensional framework of data quality. Kahn et al [19] developed a data quality framework containing conformance, completeness, and plausibility as the main data quality dimensions. This framework was the result of 2 stakeholder meetings in which data quality terms and definitions were grouped into an overall conceptual framework. The i~HD (European Institute for Innovation through Health Data) prioritized 9 data quality dimensions as most important to assess the quality of health data [20]. These dimensions were selected during a series of workshops with clinical care, clinical research, and ICT leads from 70 European hospitals. In addition, it is well known that there are several published reviews in which the results of individual quality assessment studies were collated into a new single framework of data quality dimensions. However, the results of these reviews have not yet been evaluated. Therefore, answering the “fit for purpose” question and establishing effective methods to assess data quality remain a challenge [21].

The primary objective of this review is to provide a thorough overview of data quality frameworks and their associated assessment methods, with a specific focus on the secondary use of health data, as presented in published reviews. As a secondary aim, we seek to align and consolidate the findings into a unified framework that captures the most crucial aspects of quality with a definition along with their corresponding assessment methods and requirements for testing.

## Methods

### Overview

We conducted a review of reviews to gain insights into data quality related to the secondary use of health data. In this review of reviews, we applied the Equator recommendations from the PRISMA (Preferred Reporting Items for Systematic Reviews and Meta-Analyses) guidelines proposed by Page et al [22]. As our work is primarily a review of reviews, we included only the items from these guidelines that were applicable. Abstracts were sourced by searching the PubMed, Embase, Web of Science, and SAGE databases. The search was conducted in April 2023, and only reviews published between 1995 and April 2023 were included. We used specific search terms that were aligned with the aim of our study. To ensure comprehensiveness, the search terms were expanded by searching for synonyms and relevant key terms. The following concepts were used: “data quality” or “data accuracy,” combined with “dimensions,” “quality improvement,” “data collection,” “health information interoperability,” “health information systems,” “public health information,” “quality assurance,” and “delivery of health care.” Textbox 1 illustrates an example of the search strategy used in PubMed. To ensure the completeness of the review, the literature search spanned multiple databases. All keywords and search queries were adapted and modified to suit the requirements of these various databases (Multimedia Appendix 1).

**Search query used.**

(“data quality” OR “Data Accuracy”[Mesh]) AND (dimensions OR “Quality Improvement”[Mesh] OR “Data Collection/standards”[Mesh] OR “Health Information Interoperability/standards”[Mesh] OR “Health Information Systems/standards”[Mesh] OR “Public Health Informatics/standards” OR “Quality Assurance, Health Care/standards”[Mesh] OR “Delivery of Health Care/standards”[Mesh]) Filters: Review, Systematic Review

### Inclusion and Exclusion Criteria

We included review articles that described and discussed frameworks of data quality dimensions and their assessment methods, especially from a secondary use perspective. Reviews were excluded if they were (1) not specifically related to the health care ecosystem, (2) lacked relevant information related to our research objective (no definition of dimensions), or (3) published in languages other than English.

### Selection of Articles

One reviewer (JD) screened the titles and abstracts of 982 articles from the literature searches and excluded 940 reviews. Two reviewers (RVS and JD) independently performed full-text screening of the remaining 42 reviews. Disagreements between the 2 reviewers were resolved by consulting a third reviewer (DK). After full-text screening, 20 articles were excluded because they did not meet the inclusion criteria. A total of 22 articles were included in this review.

### Data Extraction

All included articles were imported into EndNote 20 (Clarivate). Data abstraction was conducted independently by 2 reviewers (RVS and JD). Disagreements between the 2 reviewers were resolved by consulting a third reviewer (DK). The information extracted from the reviews included various details, including the authors, publication year, research objectives, specific data source used, scope of secondary use, terminology used for the data quality dimensions, their corresponding definitions, and the measurement methods used.

### Data Synthesis

To bring clarity to the diverse dimensions and definitions scattered throughout the literature, we labeled the observed definitions of dimensions from the reviews as “aspects.” We then used the framework of the i~HD. This framework underwent extensive validation through a large-scale exercise and was published [20]. It will now serve as a reference framework for mapping the diverse literature in the field. This overarching framework comprised 9 loosely delineated data quality dimensions (Textbox 2, [20]). Each observed definition of a data quality dimension was mapped onto a dimension of this reference framework. This mapping process was collaborative and required consensus among the reviewers. This consolidation is intended to offer a more coherent and unified perspective on data quality for secondary use.

**Consolidated data quality framework of the European Institute for Innovation through Health Data [20].**

**Data quality dimension and definition**

- Completeness: the extent to which data are present
- Consistency: the extent to which data satisfy constraints
- Correctness: the extent to which data are true and unbiased
- Timeliness: the extent to which data are promptly processed and up to date
- Stability: the extent to which data are comparable among sources and over time
- Contextualization: the extent to which data are annotated with acquisition context
- Representativeness: the extent to which data are representative of intended use
- Trustworthiness: the extent to which data can be trusted based on the owner’s reputation
- Uniqueness: the extent to which data are not duplicated

## Results

### Search Process

**Figure 1:**
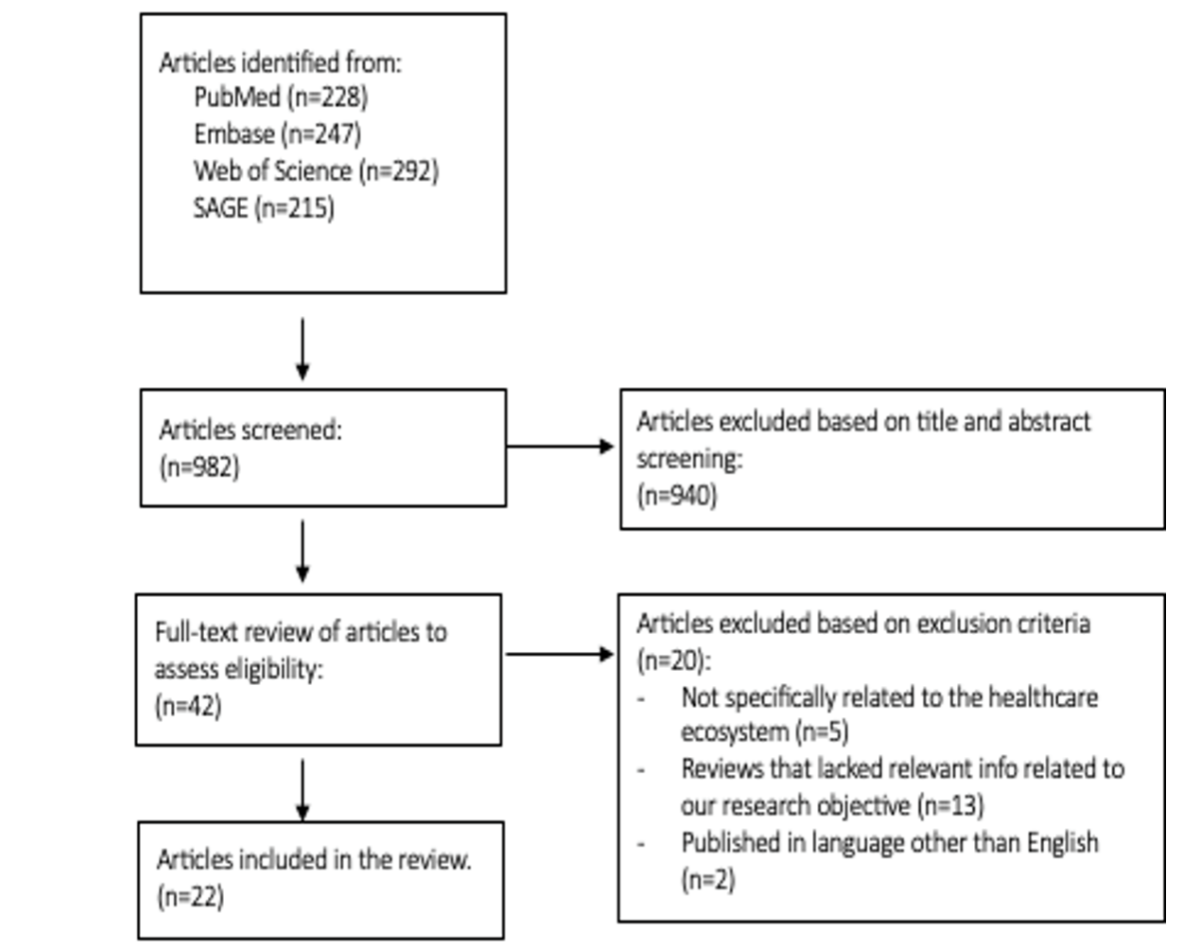
The process of selecting articles.

Figure 1 summarizes the literature review process and the articles included and excluded at every stage of the review using the PRISMA guidelines. It is important to note that this was not a systematic review of clinical trials; rather, it was an overview of existing reviews. As such, it synthesizes and analyzes the findings from multiple reviews on the topic of interest. A total of 22 articles were included in this review. The 22 reviews included systematic reviews (4/22, 18%) [23-26], scoping reviews (2/22, 9%) [27,28], and narrative reviews (16/22, 73%) [4,29-43]. All the reviews were published between 1995 and 2023. Of the 20 excluded reviews, 5 (25%) were excluded because they were not specific to the health care ecosystem [18,44-47], 13 (65%) lacked relevant information related to our research objective [6-18], and 2 (10%) were published in a language other than English [48,49].

### Data Sources

Of the 22 reviews, 10 (45%) discussed data quality pertaining to a registry [25-27,34-36,40-43] and 4 (18%) to a network of EHRs [4,24,29,33]. Of the 22 reviews, 4 (18%) discussed the quality of public health informatics systems [37,38], real-world data repositories [31], and clinical research informatics tools [30]. Of the 22 reviews, 4 (18%) did not specify their data source [23,28,32,39].

### Observed Frameworks for Data Quality Dimensions

In the initial phase of our study, we conducted a comprehensive review of 22 selected reviews, each presenting a distinct framework for understanding data quality dimensions. Across these reviews, the number of dimensions varied widely, ranging from 1 to 14 (median 4, IQR 2-5). The terminology used was diverse, yielding 23 different terms for dimensions and 62 unique definitions. A detailed overview, including data sources, data quality dimensions, and definitions, is provided in Multimedia Appendix 2 [4,23-43]. Figure S1 in Multimedia Appendix 3 presents the frequency of all dimensions in each review along with the variety of definitions associated with each dimension.

### Data Synthesis: Constructing a Consolidated Data Quality Framework For Secondary Use

#### Overview

Table 1 presents all dimensions mentioned in the included reviews, with their definitions, mapped toward each of the 9 data quality dimensions in the framework of i~HD.

**Table 1:** Mapping of data quality aspects toward i~HD (European Institute for Innovation through Health Data) data quality framework.

i~HD data quality dimensions and aspects as mentioned in the reviews		Definition
**Completeness**		
	Completeness [30,32,33,39]	The extent to which information is not missing and is of sufficient breadth and depth for the task at hand.
	Completeness [24,29,39]	This focuses on features that describe the frequencies of data attributes present in a data set without reference to data values.
	Completeness [27,35,42]	The extent to which all necessary data that could have been registered have been registered.
	Completeness [34,41]	The extent to which all the incident cases occurring in the population are included in the registry database.
	Completeness [43]	The completeness of data values can be divided between mandatory and optional data fields.
	Completeness [23]	The absence of data at a single moment over time or when measured at multiple moments over time.
	Completeness [4]	Is a truth of a patient present in the EHR^a^?
	Completeness [26]	All necessary data are provided.
	Completeness [25]	Defined as the presence of recorded data points for each variable.
	Plausibility [31]	Focuses on features that describe the frequencies of data attributes present in a data set without reference to data values.
	Capture [27,35]	The extent to which all necessary cases that could have been registered have been registered.
**Consistency**		
	Accuracy [43]	The accuracy of data values can be divided into syntactic and semantic values.
	Consistency [43]	Data inconsistencies occur when values in ≥2 data fields are in conflict.
	Consistency [39]	Representation of data values is the same in all cases.
	Consistency [26]	Data are logical across data points.
	Consistency [32]	The degree to which data have attributes that are free from contradiction and are coherent with other data in a specific content of use.
	Consistency [23]	Absence of differences between data items representing the same objects based on specific information requirements.
	Consistency [30]	Refers to the extent to which data are applicable and helpful to the task at hand.
	Correctness [26]	Data are within the specified value domains.
	Comparability [34,40]	The extent to which coding and classification procedures at a registry, together with the definitions of recoding and reporting specific data terms, adhere to the agreed international guidelines.
	Validity [30]	Refers to information that does not conform to a specific format or does not follow business rules.
	Concordance [32]	The data are concordant when there was agreement or comparability between data elements.
	Conformance [29,31]	Focuses on data quality features that describe the compliance of the representation of data against internal or external formatting, relational, or computational definitions.
	Conformance [24]	Whether the values that are present meet syntactic or structural constraints.
**Correctness**		
	Accuracy [27,35,42]	The extent to which registered data are in conformity to the truth.
	Accuracy [32,33]	The extent to which data are correct and reliable.
	Accuracy [23]	The degree to which data reveal the truth about the event being described.
	Accuracy [26]	Data conform to a verifiable source.
	Accuracy [30]	Refers to the degree to which information accurately reflects an event or object described.
	Correctness [4,24]	Is an element that is present in the EHR true?
	Correctness [39]	The free-of-error dimension.
	Plausibility [4]	Does an element in the EHR makes sense in the light of other knowledge about what that element is measuring?
	Plausibility [24]	This focuses on actual values as a representation of a real-world object or conceptual construct by examining the distribution and density of values or by comparing multiple values that have an expected relationship with each other.
	Plausibility [29]	Focuses on features that describe the believability or truthfulness of data values.
	Validity [34,40]	Defined as the proportion of cases in a data set with a given characteristic which truly have the attribute.
**Uniqueness**		
	Redundancy [32]	Data contain no redundant values.
**Stability**		
	Consistency [33]	Representations of data values remain the same in multiple data items in multiple locations.
	Consistency [24]	Refers to the consistency of data at the specified level of detail for the study’s purpose, both within individual databases and across multiple data sets.
	Currency [43]	Data currency is important for those data fields that involve information that may change over time.
	Comparability [24]	This is the similarity in data quality and availability for specific data elements used in measure across different entities, such as health plans, physicians, or data sources.
	Concordance [4,24]	Is there agreement between elements in the EHR or between the EHR and another data source?
	Information loss and degradation [24]	The loss and degradation of information content over time.
**Timeliness**		
	Timeliness [30,33,39]	The extent to which information is up to date for the task at hand.
	Timeliness [27,34,40]	Related to the rapidity at which a registry can collect, process, and report sufficiently reliable and complete data.
	Timeliness [26]	Data are available when needed.
	Currency [4]	Is an element in the EHR a relevant representation of the patient’s state at a given point in time?
	Currency [32]	The degree to which data have attributes that are of the right age in a specific context of use.
	Currency [24]	Data were considered current if they were recorded in the EHR within a reasonable period following a measurement or if they were representative of the patient’s state at a desired time of interest.
	Currency [23]	The degree to which data represent reality from the required point in time.
	Accessibility [33]	The extent to which data are available or easily and quickly retrievable.
**Contextualization**		
	Understandability [24]	The ease with which a user can understand the data.
	Understandability [30]	Refers to the degree to which the data can be comprehended.
	Contextual validity [23]	Assessment of data quality is dependent on the task at hand.
	Flexibility [24]	The extent to which data are expandable, adaptable, and easily applied to many tasks.
**Trustworthiness**		
	Security [24,39]	Personal data are not corrupted, and access is suitably controlled to ensure privacy and confidentiality.
**Representation**		
	Relevance [24,39]	The extent to which information is applicable and helpful for the task at hand.
	Precision [26]	Data value is specific.

^a^EHR: electronic health record.

#### Completeness

The first data quality dimension relates to the completeness of data. Among the 22 reviews included, 20 (91%) highlighted the significance of completeness [4,23-27,29-35,39,41-43]. Of these 20 reviews, 17 (85%) used the term completeness to refer to this dimension [4,23-27,29-35,39,41-43], whereas the remaining 3 (15%) used the terms plausibility [31] and capture [27,35].

On the basis of the definitions of completeness, we can conclude that this dimension contains 2 main aspects. First, completeness related to the data level. The most used definition related to this aspect is the extent to which information is not missing [30,32,33,39]. Other reviews focused more on features that describe the frequencies of data attributes present in a data set without reference to data values [24,29,39]. Shivasabesan et al [25], for example, defined completeness as the presence of recorded data points for each variable. A second aspect for completeness relates more to a case level, in which all the incident cases occurring in the population are included [27,34,35,41].

#### Consistency

The second data quality dimension concerns the consistency of the data. Among the 22 selected reviews, 11 (50%) highlighted the importance of consistency [23,24,26,29-32,34,39,40,43]. Although various frameworks acknowledge this as a crucial aspect of data quality, achieving a consensus on terminology and definition has proven challenging. Notably, some reviews used different terminologies to describe identical concepts associated with consistency [26,30,32,43]. Of the 11 reviews, 6 (55%) used the term consistency to describe this dimension [23,26,30,32,39,43], whereas 3 (27%) used conformance [24,29,31] and 2 (18%) referred to comparability [34,40]. Of the 11 reviews, 3 (27%) used distinct terms: accuracy [43], validity [30], and concordance [32]. Most definitions focus on data quality features that describe the compliance of the representation of data with internal or external formatting, relational, or computational definitions [29,31]. Of the 11 reviews, 2 (18%) provided a specific definition of consistency concerning registry data, concentrating on the extent to which coding and classification procedures, along with the definitions or recording and reporting of specific data terms, adhere to the agreed international guidelines [34,40]. Furthermore, Bian et al [24] concentrated on whether the values present meet syntactic or structural constraints in their definition, whereas Liaw et al [39] defined consistency as the extent to which the representation of data values is consistent across all cases.

#### Correctness

The third data quality dimension relates to the correctness of the data. Of the 22 reviews, 14 (64%) highlighted the importance of correctness [4,23,24,26,27,29,30,32-35,39,40,42]. Of the 14 reviews, 2 (14%) used 2 different dimensions to describe the same concept of correctness [4,24]. Accuracy was the most frequently used term within these frameworks [23,26,27,32,33,35,42]. In addition, other terms used included correctness [4,24,39], plausibility [4,24,29], and validity [34,40]. In general, this dimension assesses the degree to which the recorded data align with the truth [27,35,42], ensuring correctness and reliability [32,33]. Of the 14 reviews, 2 (14%) provided a specific definition of correctness concerning EHR data, emphasizing that the element collected is true [4,24]. Furthermore, of the 14 reviews, 2 (14%) defined correctness more at a data set level, defining it as the proportion of cases in a data set with a given characteristic that genuinely possess the attribute [34,40]. These reviews specifically referred to this measure as validity. Nevertheless, the use of the term validity was not consistent across the literature; it was also used to define consistency. For instance, AbuHalimeh [30] used validity to describe the degree to which information adheres to a predefined format or complies with the established business rules.

#### Timeliness

The fourth data quality dimension concerns the timeliness of the data. Among the 22 selected reviews, 11 (50%) underscored the importance of this data quality dimension [4,23,24,26,27,30,32-34,39,40]. Of the 11 reviews, 7 (64%) explicitly used the term timeliness [26,27,30,33,34,39,40], whereas 4 (36%) referred to it as currency [4,23,24,32]. Mashoufi et al [33] used the terms accessibility and timeliness to explain the same concept. Broadly, timeliness describes how promptly information is processed or how up to date the information is. Most reviews emphasized timeliness as the extent to which information is up to date for the task at hand [30,33,39]. For instance, Weiskopf and Weng [4] provided a specific definition for EHR data, stating that an element should be a relevant representation of the patient’s state at a given point in time. Other reviews defined timeliness as the speed at which data can be collected, processed, and reported [27,34,40]. Similarly, Porgo et al [26] defined timeliness as the extent to which data are available when needed.

#### Stability

The fifth data quality dimension concerns the stability of the data. Among the 22 included reviews, 4 (18%) acknowledged the significance of stability [4,24,33,43]. The most frequently used terms for this dimension are consistency [24,33] and concordance [24]. In addition, other terms used include currency [43], comparability [24], and information loss and degradation [24]. Bian et al [24] explored this aspect of data quality by using multiple terminologies to capture its multifaceted nature: stability, consistency, concordance, and information loss and degradation. This dimension, in general, encompasses 2 distinct aspects. First, it underscores the importance of data values that remain consistent across multiple sources and locations [4,24,33]. Alternatively, as described by Bian et al [24], it refers to the similarity in data quality for specific data elements used in measurements across different entities, such as health plans, physicians, or other data sources. Second, it addresses temporal changes in data that are collected over time. For instance, Lindquist [43] highlighted the importance of stability in data fields that involve information that may change over time. The term consistency is used across different data quality dimensions, but it holds different meanings depending on the context. When discussing the dimension of stability, consistency refers to the comparability of data across different sources. This ensures that information remains uniform when aggregated or compared. Compared with the consistency dimension, the term relates to the internal coherence of data within a single data set, which relates to the absence of contradiction and compliance with certain constraints. The results indicate the same ambiguity in terms of currency. When associated with stability, currency refers to the longitudinal aspect of variables. In contrast, within the dimension of timeliness, currency is concerned with the aspect if data are up to date.

#### Contextualization

The sixth data quality dimension revolves around the context of the data. Of the 22 reviews analyzed, 3 (14%) specifically addressed this aspect within their framework [23,24,30]. The most used term was understandability [24,30]. In contrast, Syed et al [23] used the term contextual validity, and Bian et al [24] referred to flexibility and understandability for defining the same concept. Broadly speaking, contextualization pertains to whether the data are annotated with their acquisition context, which is a crucial factor for the correct interpretation of results. As defined by Bian et al [24], this dimension relates to the ease with which a user can understand data. In addition, AbuHalimeh [30] refers to the degree to which data can be comprehended.

#### Representation

The seventh dimension of data quality focuses on the representation of the data. Of the 22 reviews examined, 3 (14%) specifically highlighted the importance of this dimension [24,26,39]. Of the 3 reviews, 2 (67%) used the term relevance [24,39], whereas Porgo et al [26] used the term precision. Broadly speaking, representativeness assesses whether the information is applicable and helpful for the task at hand [24,39]. In more specific terms, as defined by Porgo et al [26], representativeness relates to the extent to which data values are specific to the task at hand.

#### Trustworthiness

The eighth dimension of data quality relates to the trustworthiness of the data. Of the 22 reviews, only 2 (9%) considered this dimension in their review [24,39]. In both cases, trustworthiness was defined as the extent to which data are free from corruption, and access was appropriately controlled to ensure privacy and confidentiality.

#### Uniqueness

The final dimension of data quality relates to the uniqueness of the data. Of the 22 reviews, only 1 (5%) referred to this aspect [32]. Uniqueness is evaluated based on whether there are no duplications or redundant data present in a data set.

### Observed Data Quality Assessment Methods

#### Overview

Of the 22 selected reviews, only 8 (36%) mentioned data quality assessment methods [4,24,32,34,35,39-41]. Assessment methods were defined for 15 (65%) of the 23 data quality dimensions. The number of assessment methods per dimension ranged from 1 to 15 (median 3, IQR 1-5). There was no consensus on which method to use for assessing data quality dimensions. Figure S2 in Multimedia Appendix 3 presents the frequency of the dimensions assessed in each review, along with the number of different data quality assessment methods.

In the following section, we harmonize these assessment methods with our consolidated framework. This provides a comprehensive overview linking the assessment methods to the primary data quality dimensions from the previous section. Table 2 provides an overview of all data quality assessment techniques and their definitions. Textbox 3 presents an overview of all assessment methods mentioned in the literature and mapped toward the i~HD data quality framework.

**Table 2:** Overview of all data quality assessment methods with definitions.

Assessment M^a^	Assessment technique in reviews	Explanation
M1	Linkages—other data sets	Percentage of eligible population included in the data set.
M2	Comparison of distributions	Difference in means and other statistics.
M3	Case duplication	Number and percentage or cases with >1 record.
M4	Completeness of variables	Percentage of cases with complete observations of each variable.
M5	Completeness of cases	Percentage of cases with complete observations for all variables.
M6	Distribution comparison	Distributions or summary statistics of aggregated data from the data set are compared with the expected distributions for the clinical concepts of interest.
M7	Gold standard	A data set drawn from another source or multiple sources is used as a gold standard.
M8	Historic data methods	Stability of incidence rates over time. Comparison of incidence rates in different populations. Shape of age-specific curves. Incidence rates of childhood curves.
M9	M:I^b^	Comparing the number of deaths, sourced independently from the registry, with the number of new cases recorded for a specific period.
M10	Number of sources and notifications per case	Using many sources reduces the possibility of diagnoses going unreported, thus increasing the completeness of cases.
M11	Capture-recapture method	A statistical method using multiple independent samples to estimate the size of an entire population.
M12	Death certificate method	This method requires that death certificate cases can be explicitly identified by the data set and makes use of the M:I ratio to estimate the proportion of the initially unregistered cases.
M13	Histological verification of diagnosis	The percentage of cases morphologically verified is a measure of the completeness of the diagnostic information.
M14	Independent case ascertainment	Rescreening the sources used to detect any case missing during the registration process.
M15	Data element agreement	Two or more elements within a data set are compared to check if they report the same or compatible information.
M16	Data source agreement	Data from the data set are cross-referenced with another source to check for agreement.
M17	Conformance check	Check the uniqueness of objects that should not be duplicated; the data set agreement with prespecified or additional structural constraints, and the agreement of object concepts and formats granularity between ≥2 data sources.
M18	Element presence	A determination is made as to whether or not desired or expected data elements are present.
M19	Not specified	Number of consistent values and number of total values.
M20	International standards for classification and coding	For example, neoplasms, the International Classification of Diseases for Oncology provides coding of topography, morphology, behavior, and grade.
M21	Incidence rate	Not specified
M22	Multiple primaries	The extent that a distinction must be made between those that are new cases and those that represent an extension or recurrence of an existing one.
M23	Incidental diagnosis	Screening aims to detect cases that are asymptomatic. Autopsy diagnosis without any suspicion of diagnosed case before death.
M24	Not specified	1=ratio of violations of specific consistency type to the total number of consistency checks.
M25	Validity check	Data in the data set are assessed using various techniques that determine of the values “make sense.”
M26	Reabstracting and recoding	Reabstracting describes the process of independently reabstracting records from a given source, coding the data, and comparing the abstracted and coded data with the information recorded in the database. For each reabstracted data item, the auditor’s codes are compared with the original codes to identify discrepancies. Recoding involves independently reassigning codes to abstracted text information and evaluating the level of agreement with records already in the database.
M27	Missing information	The proportion of registered cases with unknown values for various data items.
M28	Internal consistency	The proportion of registered cases with unknown values for various data items.
M29	Domain check	Proportion of observations outside plausible range (%).
M30	Interrater variability	Proportion of observations in agreement (%). Kappa statistics.
M31	Log review	Information on the actual data entry practices (eg, dates, times, and edits) is examined.
M32	Syntactic accuracy	Not specified.
M33	Log review	Information on the actual data entry practices (eg, dates, times, and edits) is examined. Time at which data are stored in the system. Time of last update. User survey.
M34	Not specified	Ratio: number of reports sent on time divided by total reports.
M35	Not specified	Ratio: number of data values divided by the overall number of values.
M36	Time to availability	The interval between date of diagnosis (or date of incidence) and the date the case was available in the registry or data set.
M37	Security analyses	Analyses of access reports.
M38	Not specified	Descriptive qualitative measures with group interviews and interpreted with grounded theory.

^a^M: method.

^b^M:I: mortality:incidence ratio.

**Mapping of assessment methods (Ms) toward data quality framework of the European Institute for Innovation through Health Data.**

**Completeness**

- Capture [35]
  - M1: linkages—other data sets
  - M2: comparison of distributions
  - M3: case duplication
- Completeness [35]
  - M4: completeness of variables
  - M5: completeness of cases
- Completeness [32]
  - M4: completeness of variables
  - M6:distribution comparison
  - M7: gold standard
  - M5: completeness of cases
- Completeness [34]
  - M8: historic data methods
  - M9: mortality:incidence ratio (M:I)
  - M10: number of sources and notifications per case
  - M11: capture-recapture method
  - M12: death certificate method
- Completeness [41]
  - M8: historic data methods
  - M9: M:I
  - M10: number of sources and notifications per case
  - M11: capture-recapture method
  - M12: death certificate method
  - M13: histological verification of diagnosis
  - M14: independent case ascertainment
- Completeness [4]
  - M4: completeness of variables
  - M6: distribution comparison
  - M7: gold standard
  - M15: data element agreement
  - M16: data source agreement
- Completeness [24]
  - M4: completeness of variables
  - M6: distribution comparison
  - M7: gold standard
  - M17: conformance check

**Consistency**

- Conformance [24]
  - M18: element presence
  - M17: conformance check
- Concordance [32]
  - M15: data element agreement
  - M19: not specified
- Consistency [32]
  - M16: data source agreement
- Comparability [40]
  - M20: international standards for classification and coding
  - M21: incidence rate
  - M22: multiple primaries
  - M23: incidental diagnosis
  - M24: not specified
- Comparability [34]
  - M20: international standards for classification and coding
- Consistency [39]
  - M24: not specified

**Correctness**

- Correctness [4]
  - M7: gold standard
  - M15: data element agreement
- Plausibility [4]
  - M6: distribution comparison
  - M25: validity check
  - M31: log review
  - M16: data source agreement
- Validity [40]
  - M26: reabstracting and recoding
  - M13: histological verification of diagnosis
  - M27: missing information
  - M28: internal consistency
  - M12: death certificate method
- Validity [34]
  - M13: histological verification of diagnosis
  - M12: death certificate method
- Accuracy [35]
  - M7: gold standard
  - M28: internal consistency
  - M29: domain check
  - M30: interrater variability
- Correctness [24]
  - M25: validity check
- Accuracy [32]
  - M7: gold standard
  - M32: syntactic accuracy

**Stability**

- Concordance [4]
  - M15: data element agreement
  - M16: data source agreement
  - M6: distribution comparison
- Comparability [24]
  - M18: element presence
- Consistency [24]
  - M17: conformance check
- Consistency [32]
  - M15: data element agreement
  - M16: data source agreement

**Timeliness**

- Currency [32]
  - M33: log review
- Currency [4]
  - M33: log review
- Timeliness [39]
  - M34: not specified
  - M35: not specified
- Currency [24]
  - M18: element presence
- Timeliness [40]
  - M36: time to availability

**Trustworthiness**

- Security [24,39]
  - M37: security analyses

**Representation**

- Relevance [39]
  - M38: not specified

#### Completeness

Among the 20 reviews that defined data quality dimensions related to completeness, 6 (30%) incorporated data quality assessment methods into their framework [4,24,32,34,35,41]. These 6 reviews collectively introduced 17 different data quality assessment methods. Some reviews (4/6, 67%) mentioned multiple methods to evaluate completeness, which highlights the absence of a consensus within the literature regarding the most suitable approach. The most frequently used method in the literature for assessing completeness was the examination of variable completeness [4,24,32,35]. This method involved calculating the percentage of cases that had complete observations for each variable within the data set. In 3 reviews [4,24,32], researchers opted to compare the distributions or summary statistics of aggregated data from the data set with the expected distributions for the clinical concepts of interest. Another approach found in 3 reviews involved the use of a gold standard to evaluate completeness [4,24,32]. This method relied on external knowledge and entailed comparing the data set under examination with data drawn from other sources or multiple sources.

#### Consistency

Among the 15 reviews highlighting the significance of consistency, 6 (40%) defined data quality assessment methods [4,24,32,34,39,40]. In these 6 reviews, a total of 10 distinct data quality assessment methods were defined. The most used method involved calculating the ratio of violations of specific consistency types to the total number of consistency checks [32,39]. There were 2 categories established for this assessment. First, internal consistency, which focuses on the most commonly used data type, format, or label within the data set. Second, external consistency, which centered on whether data types, formats, or labels could be mapped to a relevant reference terminology or data dictionary. Another common assessment method was the implementation of international standards for classification and coding standards [34,40]. This addressed specific oncology and suggested coding for topography, morphology, behavior, and grade. Liaw et al [39] defined an assessment method in which ≥2 elements within a data set are compared to check if they report compatible information.

#### Correctness

Among the 16 reviews underscoring the importance of correctness, 6 (38%) detailed data quality assessment methods [4,24,32,34,35,40]. Collectively, these 6 reviews proposed 15 different techniques. Prominent among these were histological verification [34,40], where the percentage of morphologically verified values served as an indicator of diagnosis correctness. Another frequently used technique was the use of validity checks [4], involving various methods to assess whether the data set values “make sense.” Three additional reviews opted for a comparative approach, benchmarking data against a gold standard and calculating the sensitivity, specificity, and accuracy scores [4,32,35]. Interestingly, there is an overlap between consistency and completeness as data quality dimensions in the assessment of correctness. For instance, Weiskopf and Weng [4] defined data element agreement as an assessment for this dimension, whereas Bray and Parkin [40] evaluated the proportion of registered cases with unknown values for specific items as a correctness assessment method.

#### Stability

Among the 7 reviews emphasizing the importance of stability of the data, only 3 (43%) discussed assessment techniques that address this dimension [4,24,39]. These 3 reviews collectively outlined 5 different techniques. Notably, there was no predominant technique. Specifically, Weiskopf and Weng [4] used several techniques to assess data stability, including an overlap with other dimensions, by using data element agreement. Another technique introduced in the same review was data source agreement, involving the comparison of data from different data sets from distinct sources.

#### Timeliness

Of the 12 reviews focusing on the timeliness of data, 5 (42%) delved into assessment techniques for this data quality dimension [4,24,32,39,40]. Across these reviews, 5 distinct assessment techniques were discussed. The most commonly used technique was the use of a log review [4,39]. This method involved collecting information that provides details on data entry, the time of data storage, the last update of the data, or when the data were accessed. In addition, Bray and Parkin [40] assessed timeliness by calculating the interval between the date of diagnosis (or date of incidence) and the date the case was available in the registry or data set.

#### Trustworthiness

In the 2 reviews that considered trustworthiness as a data quality dimension, both used the same assessment technique [24,39]. This method involves the analysis of access reports as a security analysis, providing insight into the trustworthiness of the data.

#### Representation

In 1 review that addressed the representation dimension as a data quality aspect, only 1 assessment method was mentioned. Liaw et al [39] introduced descriptive qualitative measures through group interviews to determine whether the data accurately represented the intended use.

#### Uniqueness and Contextualization

No assessment methods were mentioned for these data quality dimensions.

## Discussion

### Principal Findings

This first review of reviews regarding the quality of health data for secondary use offers an overview of the frameworks of data quality dimensions and their assessment methods, as presented in published reviews. There is no consensus in the literature on the specific terminology and definitions of terms. Similarly, the methodologies used to assess these terms vary widely and are often not described in sufficient detail. Comparability, plausibility, validity, and concordance are the 4 aspects classified under different consolidated dimensions, depending on their definitions. This variability underscores the prevailing discrepancies and the urgent need for harmonized definitions. Almost none of the reviews explicitly refer to requirements of quality for the context of the data collection. Building on the insights gathered from these reviews, our consolidated framework organizes the numerous observed definitions into 9 main data quality dimensions, aiming to bring coherence to the fragmented landscape.

Health data in primary sources refer to data produced in the process of providing real-time and direct care to an individual [50], with the purpose of improving the care process. A secondary source captures data collected by someone other than the primary user and can be used for other purposes (eg, research, quality measurement, and public health) [50]. The included reviews discussed data quality for secondary use. However, the quality of health data in secondary systems is a function of the primary sources from which they originate, the quality of the process to transfer and transform the primary data to the secondary source, and the quality of the secondary source itself. The transfer and transformation of primary data to secondary sources implies the standardization, aggregation, and streamlining of health data. This can be considered as an export-transform-load (ETL) process with its own data quality implications. When discussing data quality dimensions and assessment methods, research should consider these different stages within the data life cycle, a distinction seldom made in the literature. For example, Prang et al [27] defined completeness within the context of a registry, which can be regarded as a secondary source. In this context, completeness was defined as the degree to which all potentially registrable data had been registered. The definition for completeness by Bian et al [24] pertains to an EHR, which is considered a primary source. Here, the emphasis was on describing the frequencies of data attributes. Both papers emphasized the importance of completeness, but they approached this dimension from different perspectives within the data life cycle.

This fragmented landscape regarding terminology and definition of data quality dimensions, the lack of distinction between quality in primary and secondary data and in the ETL process, and the lack of consideration for the context allows room for interpretation, leading to difficulties in developing assessment methods. In our included articles, only 8 (36%) out of 22 reviews mentioned and defined assessment methods [4,24,32,34,35,39-41]. However, the results showed that the described assessment methods are limited by a lack of well-defined and standardized metrics that can quantitatively or qualitatively measure the quality of data across various dimensions and often suffer from inadequate translation of these dimensions into explicit requirements for primary and secondary data and the ETL process, considering the purpose of the data collection of the secondary source. Both the DAMA and ISO emphasize in their definition of data quality that requirements serve as the translation of dimensions. Data quality dimensions refer to a broad context or characteristics of data that are used to assess the quality of data. Data quality requirements are derived from data quality dimensions and specify the specific criteria or standards that data must meet to be considered high-quality data. These requirements define the specific thresholds that need to be achieved for each dimension. However, our results show that the focus of the literature lies in defining dimensions and frameworks, rather than adequately developing these essential data quality requirements.

To avoid further problems and ambiguities, it is important to understand the purpose, context, and limitations of the data and data sources to establish a comprehensive view on the quality of the data. Rather than pursuing an elusive quest in the literature for a rigid framework defined by a fixed number of dimensions and precise definitions, future research should shift its focus toward defining and developing specific data quality requirements tailored to each use case. This approach should consider various stages within the data life cycle. For example, when defining a specific completeness requirement for a secondary use case, it will impact the way data are generated at the primary source and how they are transformed and transferred between the primary and secondary sources. Creating explicit requirements that align with the purpose of each use case along with well-defined criteria and thresholds can foster the development of precise assessment methods for each dimension. Moreover, formulating these use case requirements will facilitate addressing the fundamental question of whether health data are fit for purpose, thus determining if they are of a sufficient quality.

### Limitations

The strength of a review of reviews methodology is to provide a comprehensive overview of the current state of knowledge. However, it is important to acknowledge that this approach may have limitations, particularly in identifying new studies that have not yet undergone review or inclusion in the existing body of literature. Terms such as “information quality,” “error check,” “data check,” “data validation,” and “data cleaning” are commonly associated with the concept of data quality, particularly in older research papers. However, we did not include these terms in our search query because subsequent checking using these terms did not reveal any additional reviews that met our inclusion criteria. Furthermore, this overview focused on published reviews. Important information can also be found in grey literature [51,52] and in studies that collect stakeholders’ opinions on the quality of health data [20]. Finally, none of the included reviews discussed patient-generated data or data generated by wearables. Given the increasing adoption and use of these sources in health care, it is becoming important to consider their impact on data quality. Developing assessment methods that are applicable to these emerging data sources is an important area for further research.

Although having a consolidated reference framework of data quality dimensions and aspects is valuable, it is also of great importance to define specific data quality requirements for each relevant aspect within a single quality dimension. These requirements should specify the desired quality level to be achieved in a given percentage of the primary sources, based on the purpose of the data collection or a particular real-world data study. Once these requirements are clearly articulated, appropriate measurement methods can be determined, thereby ensuring the comprehensive analysis of secondary data collection for its suitability for a specific purpose.

### Conclusions

The absence of a consensus in the literature regarding the precise terminology and definitions of data quality dimensions has resulted in ambiguity and challenges in creating specific assessment methods. This review of reviews offers an overview of data quality dimensions, along with the definitions and assessment methods used in these reviews. This study goes a step further by assigning all observed definitions to a consolidated framework of 9 data quality dimensions. Further research is needed to complete the collection of aspects within each quality dimension, with the elaboration of a full set of assessment methods, and the establishment of specific requirements to evaluate the suitability for the purpose of secondary data collection systems.

## Appendix

Multimedia Appendix 1 Search items by database.

Multimedia Appendix 2 Data sources, data quality aspects, and definitions reported in the 22 publications included in the review.

Multimedia Appendix 3 The frequency of all dimensions with definitions in each review and assessment methods per dimension.

Multimedia Appendix 4 PRISMA (Preferred Reporting Items for Systematic Reviews and Meta-Analyses) checklist.

## Abbreviations

EHR: electronic health record
ETL: export-transform-load
i~HD: European Institute for Innovation through Health Data
ISO: International Organization for Standardization
PRISMA: Preferred Reporting Items for Systematic Reviews and Meta-Analyses

## References

[1] Duncan R, Eden R, Woods L, Wong I, Sullivan C (2022). Synthesizing dimensions of digital maturity in hospitals: systematic review. J Med Internet Res.

[2] Eden R, Burton-Jones A, Scott I, Staib A, Sullivan C (2018). Effects of eHealth on hospital practice: synthesis of the current literature. Aust Health Rev.

[3] Zheng K, Abraham J, Novak LL, Reynolds TL, Gettinger A (2018). A survey of the literature on unintended consequences associated with health information technology: 2014–2015. Yearb Med Inform.

[4] Weiskopf NG, Weng C (2013). Methods and dimensions of electronic health record data quality assessment: enabling reuse for clinical research. J Am Med Inform Assoc.

[5] Feder SL (2018). Data quality in electronic health records research: quality domains and assessment methods. West J Nurs Res.

[6] Bell SK, Delbanco T, Elmore JG, Fitzgerald PS, Fossa A, Harcourt K, Leveille SG, Payne TH, Stametz RA, Walker J, DesRoches CM (2020). Frequency and types of patient-reported errors in electronic health record ambulatory care notes. JAMA Netw Open.

[7] Weir CR, Hurdle JF, Felgar MA, Hoffman JM, Roth B, Nebeker JR (2003). Direct text entry in electronic progress notes. An evaluation of input errors. Methods Inf Med.

[8] Suresh G (2003). Don't believe everything you read in the patient's chart. Pediatrics.

[9] Kaboli PJ, McClimon BJ, Hoth AB, Barnett MJ (2004). Assessing the accuracy of computerized medication histories. Am J Manag Care.

[10] Staroselsky M, Volk LA, Tsurikova R, Newmark LP, Lippincott M, Litvak I, Kittler A, Wang T, Wald J, Bates DW (2008). An effort to improve electronic health record medication list accuracy between visits: patients' and physicians' response. Int J Med Inform.

[11] Yadav S, Kazanji N, Paudel S, Falatko J, Shoichet S, Maddens M, Barnes MA, Narayan KC (2017). Comparison of accuracy of physical examination findings in initial progress notes between paper charts and a newly implemented electronic health record. J Am Med Inform Assoc.

[12] Darko-Yawson S, Ellingsen G (2016). Assessing and improving EHRs data quality through a socio-technical approach. Procedia Comput Sci.

[13] Wang Z, Penning M, Zozus M (2019). Analysis of anesthesia screens for rule-based data quality assessment opportunities. Stud Health Technol Inform.

[14] Puttkammer N, Baseman JG, Devine EB, Valles JS, Hyppolite N, Garilus F, Honoré JG, Matheson AI, Zeliadt S, Yuhas K, Sherr K, Cadet JR, Zamor G, Pierre E, Barnhart S (2016). An assessment of data quality in a multi-site electronic medical record system in Haiti. Int J Med Inform.

[15] Wiebe N, Xu Y, Shaheen AA, Eastwood C, Boussat B, Quan H (2020). Indicators of missing Electronic Medical Record (EMR) discharge summaries: a retrospective study on Canadian data. Int J Popul Data Sci.

[16] von Lucadou M, Ganslandt T, Prokosch HU, Toddenroth D (2019). Feasibility analysis of conducting observational studies with the electronic health record. BMC Med Inform Decis Mak.

[17] Juran JM, Gryna FM, Bingham RS (1974). Quality Control Handbook.

[18] Ehrlinger L, Wöß W (2022). A survey of data quality measurement and monitoring tools. Front Big Data.

[19] Kahn MG, Callahan TJ, Barnard J, Bauck AE, Brown J, Davidson BN, Estiri H, Goerg C, Holve E, Johnson SG, Liaw S, Hamilton-Lopez M, Meeker D, Ong TC, Ryan P, Shang N, Weiskopf NG, Weng C, Zozus MN, Schilling L (2016). A harmonized data quality assessment terminology and framework for the secondary use of electronic health record data. EGEMS (Wash DC).

[20] Aerts H, Kalra D, Saez C, Ramírez-Anguita JM, Mayer MA, Garcia-Gomez JM, Hernández MD, Thienpont G, Coorevits P (2021). Is the quality of hospital EHR data sufficient to evidence its ICHOM outcomes performance in heart failure? A pilot evaluation. medRxiv. Preprint posted online February 5, 2021.

[21] Ge M, Helfert M (2007). A review of information quality research - develop a research agenda. Proceedings of the 2007 MIT International Conference on Information Quality.

[22] Page MJ, McKenzie JE, Bossuyt PM, Boutron I, Hoffmann TC, Mulrow CD, Shamseer L, Tetzlaff JM, Akl EA, Brennan SE, Chou R, Glanville J, Grimshaw JM, Hróbjartsson A, Lalu MM, Li T, Loder EW, Mayo-Wilson E, McDonald S, McGuinness LA, Stewart LA, Thomas J, Tricco AC, Welch VA, Whiting P, Moher D (2021). The PRISMA 2020 statement: an updated guideline for reporting systematic reviews. BMJ.

[23] Syed R, Eden R, Makasi T, Chukwudi I, Mamudu A, Kamalpour M, Kapugama Geeganage D, Sadeghianasl S, Leemans SJ, Goel K, Andrews R, Wynn MT, Ter Hofstede A, Myers T (2023). Digital health data quality issues: systematic review. J Med Internet Res.

[24] Bian J, Lyu T, Loiacono A, Viramontes TM, Lipori G, Guo Y, Wu Y, Prosperi M, George TJ, Harle CA, Shenkman EA, Hogan W (2020). Assessing the practice of data quality evaluation in a national clinical data research network through a systematic scoping review in the era of real-world data. J Am Med Inform Assoc.

[25] Shivasabesan G, Mitra B, O'Reilly GM (2018). Missing data in trauma registries: a systematic review. Injury.

[26] Porgo TV, Moore L, Tardif PA (2016). Evidence of data quality in trauma registries: a systematic review. J Trauma Acute Care Surg.

[27] Prang KH, Karanatsios B, Verbunt E, Wong HL, Yeung J, Kelaher M, Gibbs P (2022). Clinical registries data quality attributes to support registry-based randomised controlled trials: a scoping review. Contemp Clin Trials.

[28] Nesca M, Katz A, Leung C, Lix L (2022). A scoping review of preprocessing methods for unstructured text data to assess data quality. Int J Popul Data Sci.

[29] Ozonze O, Scott PJ, Hopgood AA (2023). Automating electronic health record data quality assessment. J Med Syst.

[30] AbuHalimeh A (2022). Improving data quality in clinical research informatics tools. Front Big Data.

[31] Liaw S, Guo JG, Ansari S, Jonnagaddala J, Godinho MA, Borelli AJ, de Lusignan S, Capurro D, Liyanage H, Bhattal N, Bennett V, Chan J, Kahn MG (2021). Quality assessment of real-world data repositories across the data life cycle: a literature review. J Am Med Inform Assoc.

[32] Rajan NS, Gouripeddi R, Mo P, Madsen RK, Facelli JC (2019). Towards a content agnostic computable knowledge repository for data quality assessment. Comput Methods Programs Biomed.

[33] Mashoufi M, Ayatollahi H, Khorasani-Zavareh D (2018). A review of data quality assessment in emergency medical services. Open Med Inform J.

[34] Fung JW, Lim SBL, Zheng H, Ho WY, Lee BG, Chow KY, Lee HP (2016). Data quality at the Singapore cancer registry: an overview of comparability, completeness, validity and timeliness. Cancer Epidemiol.

[35] O'Reilly GM, Gabbe B, Moore L, Cameron PA (2016). Classifying, measuring and improving the quality of data in trauma registries: a review of the literature. Injury.

[36] Stausberg J, Nasseh D, Nonnemacher M (2015). Measuring data quality: a review of the literature between 2005 and 2013. Stud Health Technol Inform.

[37] Chen H, Yu P, Hailey D, Wang N (2014). Methods for assessing the quality of data in public health information systems: a critical review. Stud Health Technol Inform.

[38] Chen H, Hailey D, Wang N, Yu P (2014). A review of data quality assessment methods for public health information systems. Int J Environ Res Public Health.

[39] Liaw ST, Rahimi A, Ray P, Taggart J, Dennis S, de Lusignan S, Jalaludin B, Yeo A, Talaei-Khoei A (2013). Towards an ontology for data quality in integrated chronic disease management: a realist review of the literature. Int J Med Inform.

[40] Bray F, Parkin DM (2009). Evaluation of data quality in the cancer registry: principles and methods. Part I: comparability, validity and timeliness. Eur J Cancer.

[41] Parkin DM, Bray F (2009). Evaluation of data quality in the cancer registry: principles and methods Part II. Completeness. Eur J Cancer.

[42] Arts DG, De Keizer NF, Scheffer GJ (2002). Defining and improving data quality in medical registries: a literature review, case study, and generic framework. J Am Med Inform Assoc.

[43] Lindquist M (2004). Data quality management in pharmacovigilance. Drug Saf.

[44] Haug A (2021). Understanding the differences across data quality classifications: a literature review and guidelines for future research. Ind Manag Data Syst.

[45] Triki Z, Bshary R (2022). A proposal to enhance data quality and FAIRness. Ethol.

[46] Šlibar B, Oreški D, Begičević Ređep NB (2021). Importance of the open data assessment: an insight into the (meta) data quality dimensions. SAGE Open.

[47] Verma R (2012). Data quality and clinical audit. Intensive Care Med.

[48] Lima CR, Schramm JM, Coeli CM, da Silva ME (2009). [Review of data quality dimensions and applied methods in the evaluation of health information systems]. Cad Saude Publica.

[49] Correia LO, Padilha BM, Vasconcelos SM (2014). [Methods for assessing the completeness of data in health information systems in Brazil: a systematic review]. Cien Saude Colet.

[50] Safran C, Bloomrosen M, Hammond WE, Labkoff S, Markel-Fox S, Tang PC, Detmer DE, Expert Panel (2007). Toward a national framework for the secondary use of health data: an American Medical Informatics Association white paper. J Am Med Inform Assoc.

[51] (2022). European health data space data quality framework. European Union's 3rd Health Programme.

[52] (2023). Data quality framework for EU medicines regulation. European Medicines Agency.

