# Supplementary material for: Frameworks, Dimensions, Definitions of Aspects, and Assessment Methods for the Appraisal of Quality of Health Data for Secondary Use: Comprehensive Overview of Reviews
Source: JMIR Med Inform. 2024 Mar 6;12:e51560. doi: 10.2196/51560 (PMC10955383; doi:10.2196/51560)
Supplement: Multimedia Appendix 1 [file medinform_v12i1e51560_app1.docx]

**Appendix 1. Search items by database**

| Database | Search query |
| --- | --- |
| PubMed | ("data quality" OR "Data Accuracy"[Mesh]) AND (dimensions OR "Quality Improvement"[Mesh] OR "Data Collection/standards"[Mesh] OR "Health Information Interoperability/standards"[Mesh] OR "Health Information Systems/standards"[Mesh] OR "Public Health Informatics/standards" OR "Quality Assurance, Health Care/standards"[Mesh] OR "Delivery of Health Care/standards"[Mesh]) Filters: Review, Systematic Review, from 1995 - 2023 |
| Embase | ('data quality' OR 'data accuracy/exp') AND ((dimensions OR 'quality improvement/exp' OR data) AND collection OR 'data interoperability/exp' OR 'medical information system/exp' OR 'medical informatics/exp' OR 'health care quality/exp' OR 'health care delivery/exp') AND (1995:py OR 1996:py OR 1997:py OR 1998:py OR 1999:py OR 2000:py OR 2001:py OR 2002:py OR 2003:py OR 2004:py OR 2005:py OR 2006:py OR 2007:py OR 2008:py OR 2009:py OR 2010:py OR 2011:py OR 2012:py OR 2013:py OR 2014:py OR 2015:py OR 2016:py OR 2017:py OR 2018:py OR 2019:py OR 2020:py OR 2021:py OR 2022:py OR 2023:py) AND 'review'/it |
| Web of Science | Results for ("data quality" OR "Data Accuracy") AND ("dimensions" OR "Quality Improvement" OR "Data Collection" OR "Health Information Interoperability" OR "Health Information Systems" OR "Public Health Informatics" OR "health quality assurance" OR "Delivery of Health Care") (All Fields) and Review Article (Document Types) and 2023 or 2022 or 2021 or 2020 or 2019 or 2018 or 2017 or 2016 or 2015 or 2013 or 2014 or 2012 or 2011 or 2010 or 2009 or 2008 or 2007 or 2006 or 2005 or 2004 or 2003 or 2002 or 2001 or 2000 or 1999 or 1998 or 1997 or 1996 or 1995 (Publication Years) |
| SAGE | ("data quality" OR "Data Accuracy") AND (dimensions OR "Quality Improvement" OR "Data Collection/standards" OR "Health Information Interoperability/standards" OR "Health Information Systems/standards" OR "Public Health Informatics/standards" OR "Quality Assurance, Health Care/standards" OR "Delivery of Health Care/standards") Filters: Review article, from 1995 - 2023 |
