# Supplementary material for: Frameworks, Dimensions, Definitions of Aspects, and Assessment Methods for the Appraisal of Quality of Health Data for Secondary Use: Comprehensive Overview of Reviews
Source: JMIR Med Inform. 2024 Mar 6;12:e51560. doi: 10.2196/51560 (PMC10955383; doi:10.2196/51560)
Supplement: Multimedia Appendix 2 [file medinform_v12i1e51560_app2.docx]

| Author and year | Study objective | Data source | Data quality aspect | Data quality definition | Conclusions |
| --- | --- | --- | --- | --- | --- |
| Arts *et al.* 2002 [42] | To identify causes of insufficient data quality and to make a list of procedures for data quality assurance in medical registries and put them in a framework. | Medical registry | Accuracy | The extent to which registered data are in conformity to the truth. | Definitions of data quality and data quality attributes in literature are unclear, ambiguous, or unavailable. Standard definitions of data quality and attributes are necessary to be able to compare data quality among registries or within a registry at different points in time. |
|  |  |  | Completeness | The extent to which all necessary data that could have been registered have been registered. |  |
| Lindquist *et al.* 2004 [43] | To provide an overview of data quality management in pharmacovigilance. | Registry | Accuracy | The accuracy of data values can be divided into syntactic and semantic values. | There are several data quality dimensions involving data processing. The maintenance of good quality must be upheld throughout the processing cycle, and in the computerized systems employed as supporting platforms. The aim should be to have transparent, well documented, and functioning quality systems in place. |
|  |  |  | Completeness | The completeness of data values can be divided between mandatory and optional data fields. |  |
|  |  |  | Consistency | Data inconsistencies occur when values in two or more data fields are in conflict. |  |
|  |  |  | Currency | Data currency is important for those data fields that involve information that may change over time. |  |
| Bray *et al.* 2008 [40] | To provide an update of the practical aspects and techniques for addressing data quality at the cancer registry (part I). | Cancer registry | Comparability | The extent to which coding and classification procedures at a registry, together with the definitions of recording and reporting specific data items, adhere to agreed international guidelines. | Not specified |
|  |  |  | Validity | Defined as the proportion of cases in a dataset with a given characteristic which truly have the attribute. |  |
|  |  |  | Timeliness | Relates to the rapidity at which a registry can collect, process, and report sufficiently reliable and complete data. |  |
| Parkin *et al.* 2009 [41] | To provide an update of the practical aspects and techniques for addressing data quality at the cancer registry (part II). | Cancer registry | Completeness | The extent to which all of the incident cases occurring in the population are included in the registry database. | Not specified |
| Weiskopf *et al.* 2013 [4] | To review the methods and dimensions of data quality assessment in the context of electronic health record data reuse for research. | Electronic health record | Completeness | Is a truth of a patient present in the EHR? | There is little consistency of potential generalizability in the methods used to assess EHR data quality. If the reuse of EHR data for clinical research is to become accepted, researchers should adopt validated, systematic methods of EHR data quality assessment. |
|  |  |  | Correctness | Is an element that is present in the EHR true? |  |
|  |  |  | Concordance | Is there agreement between elements in the EHR, or between the EHR and another data source? |  |
|  |  |  | Plausibility | Does an element in the EHR makes sense in light of other knowledge about what that element is measuring? |  |
|  |  |  | Currency | Is an element in the EHR a relevant representation of the patient state at a given point in time? |  |

| Liaw *et al.* 2013 [39] | What is/are the role(s) of ontologies in the assessment and management of data quality to support better decision making and measurement of health outcomes in integrated chronic disease management (CDW)? | Not specified | Completeness | - The extent to which information is not missing and is of sufficient breadth and depth for the task at hand. - The ability of an information system to represent every meaningful state of the represented real-world system. - Degree to which information is sufficient to depict every possible state of the task. - All values for a variable are recorded. - Availability or defined minimum number of records/patients. | This literature review was limited by the immaturity of the field. The challenges for the development and validation of a data quality ontology in chronic disease management include.   - Methodological immaturity. - An immature knowledge base. - A lack of tools to support ontology-based database design for clinical data warehouses. - Evaluation of ontological approaches. - Engagement of users in design and implementations. |
| --- | --- | --- | --- | --- | --- |
|  |  |  | Consistency | - Representation of data values is same in all cases. - Includes values and physical representation of data. - The extent to which information is easy to manipulate and apply to different tasks. - The equivalence, and process to achieve, equivalence of information stored or used in applications, and systems. - The extent of use of a uniform data type and format with a uniform data label (internal consistency) and codes/terms that can be mapped to a reference terminology (external consistency). |  |
|  |  |  | Correctness | - The free-of-error dimension. - Credibility of source and user’s level of expertise. - Data values, format and types are valid and appropriate. |  |
|  |  |  | *Accuracy* | Recorded value is in conformity with actual value. |  |
|  |  |  | *Reliability* | - Extent to which a data can be expected to perform its intended function with required/defined accuracy. - How data conforms with user requirements or reality. - Data can be counted on to convey the right information. |  |
|  |  |  | Timeliness | - Data is not out of date; availability of output is on time. - Extent to which information is up to date for task. - The delay between a change of the real-world state and the resulting modification of the information system state. |  |
|  |  |  | Relevance | The extent to which information is applicable and helpful for the task at hand. |  |
|  |  |  | Usability | The degree to which data can be accessed, used, updated, maintained, managed to enable effective decisions. |  |
|  |  |  | Security | Personal data is not completed, and access suitably controlled to ensure privacy and confidentiality. |  |

| Chen *et al.* 2014 [37] | To investigate and compare the methods for data quality assessment of public health information systems. | Public health information systems | Completeness | Not specified | The results suggest that the theory of measurement has been applied either explicitly or implicitly in the development of data quality assessment methods for PHIS.  Qualitative data quality assessment primarily used descriptive surveys and data audits, while qualitative data quality assessment methods include primarily interview, documentation review and field observation. |
| --- | --- | --- | --- | --- | --- |
|  |  |  | Accuracy | Not specified |  |
|  |  |  | Timeliness | Not specified |  |
| Chen *et al.* 2014 [38] | To evaluate data quality assessment methods. Focus on which attributes were assessed, how the assessment was conducted, and the reliability and validity of each method. | Public health information systems | Completeness | Not specified | The results of this review demonstrate there is plenty of room for further development of effective data quality assessment methods in the context of public health.Stausber |
|  |  |  | Accuracy | Not specified |  |
|  |  |  | Timeliness | Not specified |  |
| Stausberg *et al.* 2015 [36] | To provide a review of the literature about data quality from 2005 to 2013. | Medical registry | Case completeness (completeness) | Not specified | Information about the quality of medical data should be regarded as an obligatory supplement of data pools. This review offers several candidate measures. |
|  |  |  | Data completeness (comprehensive) | Not specified |  |
|  |  |  | Correctness | Not specified |  |
| O’Reilly *et al.* 2016 [35] | To review the accessible literature to determine the methods used to classify, measure, and improve data quality in trauma registry. | Trauma registry | Accuracy | The extent to which registered data are in conformity with the truth. | There is a clear need for additional research and a globally accessible document standardizing the definition, classification measurement, improvement, and reporting of data quality in trauma registry. |
|  |  |  | Completeness | The extent to which all necessary data have been registered on registered cases. |  |
|  |  |  | Capture | The extent to which all necessary cases that could have been registered have been registered. |  |

| Porgo *et al.* 2016 [26] | To review evidence of completeness, accuracy, precision, correctness, consistency, and timeliness of trauma registry data. | Trauma registry | Completeness | All necessary data are provided. | Few studies have evaluated data quality in trauma registries, and evaluation of data quality is mostly based on completeness. There is a need to evaluate methods to evaluate data quality based on all data quality dimensions. |
| --- | --- | --- | --- | --- | --- |
|  |  |  | Accuracy | Data conform with a verifiable source. |  |
|  |  |  | Precision | Data value is specific. |  |
|  |  |  | Correctness | Data are within specified value domains. |  |
|  |  |  | Consistency | Data are logical across data points. |  |
|  |  |  | Timeliness | Trauma registry data are available when needed. |  |
| Fung *et al.* 2016 [34] | To present the first comprehensive evaluation of data quality at the Singapore cancer registry. | Singapore cancer registry (SRC) | Comparability | The extent of which coding and collection practices adhere to international guidelines. | This review indicated that the SCR adheres to international guidelines, has a high degree of completeness, accuracy, and timeliness. It should however be kept in mind that an efficient national registry system is not the end goal. The eventual objectives are to step up actions to prevent, control, and improve interventions for cancer care. |
|  |  |  | Completeness | The extent to which all diagnosed cancer cases in Singapore are captured in the registry. |  |
|  |  |  | Validity | The proportion of cases in the registry with a given characteristics which truly have this attribute. |  |
|  |  |  | Timeliness | Using the median time taken from diagnosis to registration and the time from registration to the reporting of incidence of SCR’s annual reports. |  |
| Shivasabesan *et al.* 2018 [25] | To examine the current practice of trauma registry-based studies regarding the identification, reporting, and management of missing data. | Trauma registry | Completeness | Defined as the presence of recorded data points for each variable. | There is a lack of standardization in the reporting and management of missing data questions the validity of conclusions from research based on trauma registry data. |
| Mashoufi *et al.* 2018 [33] | To identify the assessment approaches, the main dimensions of data quality and the status of data quality in the emergency medical services. | Emergency medical services | Accuracy | The extent to which data is correct and reliable. | The results showed that due to the diversity of definitions and terminology used to assess dimensions, characteristics, and attributes of data quality, comparing findings reported in different studies was difficult. Therefore more attention should be paid to choose a clear and a consistent definition of data quality. |
|  |  |  | Completeness | The extent to which data is not missing and is sufficient for the task at hand. |  |
|  |  |  | Timeliness | The extent to which data is sufficiently up to date for the task at hand. |  |
|  |  |  | Accessibility | The extent to which data is available, or easily and quickly retrievable. |  |
|  |  |  | Consistency | Representations of data values remains the same in multiple data items in multiple locations. |  |

| Bian *et al.* 2020 [24] | To synthesize data quality dimensions and assessment methods of real-world data, through a systematic scoping review and to assess the practice of data quality assessment in the national Patient-centered Clinical Research Network (PCORnet). | Electronic health record | Currency | Data were considered current if they were recorded in the EHR within a reasonable period of time following a measurement or if they were representative of the patient state at a desired time of interest. | Definitions of data quality dimensions and methods were not consistent in the literature, and the data quality assessment practice was not evenly distributed. Challenges in data quality assessments, given the complex and heterogeneous nature of real-world data, exist. |
| --- | --- | --- | --- | --- | --- |
|  |  |  | Correctness | EHR data were considered correct when the information they contained was true. |  |
|  |  |  | Plausibility | This focuses on actual values as a representation of a real-world object or conceptual construct by examining the distribution and density of values or by comparing multiple values that have an expected relationship to each other. |  |
|  |  |  | *Uniqueness* | This subcategory seeks to determine if objects appear multiple times in settings where they should not be duplicated or cannot be distinguished within a database or when compared with an external reference. |  |
|  |  |  | *Atemporal* | This subcategory seeks to determine if observed data values agree with local or “common” knowledge or from comparisons with external sources that are deemed to be trusted or relative gold standard. |  |
|  |  |  | *Temporal* | This subcategory seeks to determine of time-varying variables change values as expected based on known temporal properties or across 1 or more external comparators or gold standards. |  |
|  |  |  | Completeness | This focuses on features that describe the frequencies of data attributes present in a data set without reference to data values. |  |
|  |  |  | Concordance | Is there agreement between elements in the EHR, or between the EHR and another data source? |  |
|  |  |  | Comparability | This is the similarity in data quality and availability for specific data elements used in measure across different entities, such as health plans or physicians or data sources. |  |
|  |  |  | Conformance | Whether the values that are present meet syntactic or structural constraints. |  |
|  |  |  | *Value* | Agreement with a prespecified, constraint-driven data architecture |  |
|  |  |  | *Relational* | Agreement with additional structural constraints imposed by the physical database structures that store data values. |  |
|  |  |  | *Computational* | If computations used to create derived values from existing variables yield the intended results either within a data set or between data sets, when programs are based on identical specifications. |  |
|  |  |  | Flexibility | The extent to which data are expandable, adaptable, and easily applied to many tasks. |  |

|  |  |  | Relevance | The extent to which information is applicable and helpful for the task at hand. |  |
| --- | --- | --- | --- | --- | --- |
|  |  |  | Usability/Easy-of-Use | A measure of the degree to which data can be accessed and used and the degree to which data can be updated, maintained, and managed. |  |
|  |  |  | Security | Personal data is not corrupted, and access suitable controlled to ensure privacy and confidentiality. |  |
|  |  |  | Information Loss and Degradation | The loss and degradation of information content over time. |  |
|  |  |  | Consistency | Pertains to the constancy of the data, at the desired degree of detail for the study purpose, within, and across databases and data sets. |  |
|  |  |  | Understandability/ Interpretability | The ease with which a user can understand the data. |  |
| Liaw *et al.* 2021 [31] | To complete a literature review on data quality assessment frameworks, indicators and tools for research, public health, service, and quality improvement across the data life cycle. | Real world data repositories | Conformance | Focuses on data quality features that describe the compliance of the representation of data against internal or external formatting, relational, or computational definitions. | meso-, and macro-organizations as well as the health system across the data life cycle.  The enhanced data quality assessment framework in this review will determine fitness for purpose through assessment of intrinsic, contextual, and technical data quality. |
|  |  |  | *Value* | This seeks to determine of recorded data elements are in agreement with a prespecified, constraint – driven data architecture. |  |
|  |  |  | *Relational* | This seeks to determine if recorded data elements are in agreement with additional structural constraints imposed by the physical database structure that store data values. |  |
|  |  |  | *Computational* | This seeks to determine if computations used to create derived values from existing variables yield the intended results either within a data set or between data sets, when programs are based on identical specifications. |  |
|  |  |  | Completeness | Focuses on features that describe the frequencies of data attributes present in a data set without reference to data values. |  |
|  |  |  | Plausibility | Focuses on features that describes the believability or truthfulness of data values. |  |
|  |  |  | *Uniqueness* | This seeks to determine of objects appear multiple times in settings where they should not be duplicated or cannot be distinguished within a database or when compared with an external reference. |  |
|  |  |  | *Atemporal* | This seeks to determine if observed data values, distributions, or densities agree with local or common knowledge or from comparisons with external sources that are deemed to be trusted or relative gold standards |  |
|  |  |  | *Temporal* | This seeks to determine of time-varying variables change values as expected based on known temporal properties or across 1 or more external comparators or gold standards. |  |
|  |  |  | Data Organisation | Not specified |  |
|  |  |  | *Timeliness* | Not specified |  |
|  |  |  | *Trust* | Not specified |  |
|  |  |  | *Relevance* | Not specified |  |
|  |  |  | *Accessibility* | Not specified |  |
|  |  |  | *Reusability* | Not specified |  |
|  |  |  | *Governance* | Not specified |  |
|  |  |  | *Technical* | Not specified |  |
|  |  |  | *Traceability* | Not specified |  |
|  |  |  | *Interoperability* | Not specified |  |

|  |  |  |  |
| --- | --- | --- | --- |
|  |  |  |  |
|  |  |  |  |
|  |  |  |  |

| Rajan *et al.* 2019 [32] | To investigate an agnostic approach for data quality assessment, and to design and implement a computable quality knowledge repository capable of storing any data quality framework and their methods of computation. | N/A | Accuracy | The extent to which data are correct, reliable, and certified free of errors. | The results identified research gaps in data quality literature towards automating data quality assessment methods. In this process, the researchers designed, developed, and implemented a computable data quality knowledge repository for assessing quality and characterizing data in health data repositories. |
| --- | --- | --- | --- | --- | --- |
|  |  |  | Completeness | The extent to which data are of sufficient breadth, depth, and scope for the task at hand. |  |
|  |  |  | Concordance | The data is concordant when there was agreement or comparability between data elements. |  |
|  |  |  | Consistency | The degree to which data has attributes that are free from contradiction and are coherent with other data in a specific content of use. |  |
|  |  |  | Currency | The degree to which data has attributes that are of the right age in a specific context of use. |  |
|  |  |  | Redundancy | Data contains no redundant values. |  |

| AbuHalimeh *et al.* 2022 [30] | To discuss the quality of data generated from two systems, understand the potential causes of the data quality issues, and propose steps to improve the data quality. | Clinical research informatics tools | Accuracy | Refers to the degree to which information accurately reflects an event or object described. | This review proved that the discrepancies in the data used in the data pull process led to major information quality issues such as data inconsistency and data accuracy which both affects the believability and the validity of the data which also are major data quality measures. |
| --- | --- | --- | --- | --- | --- |
|  |  |  | Completeness | Refers to the extent to which data is not missing and of sufficient amount for the task at hand. |  |
|  |  |  | Consistency | Refers to the extent to which data is applicable and helpful to the task at hand. |  |
|  |  |  | Timeliness | Refers to the extent to which data is sufficiently up to date for the task at hand. |  |
|  |  |  | Validity | Refers to information that doesn’t conform to a specific format or doesn’t follow business rules. |  |
|  |  |  | Understandability | Refers to the degree the data can be comprehended |  |
| Nesca *et al.* 2022 [28] | To systematically document current research and practices about natural language preprocessing methods for unstructured text data to describe or improve its quality. | N/A | Accuracy | N/A | Assessing data quality in unstructured text data often requires access to specialized gold standard datasets or dictionaries. However, there are a few general-purpose measures of data quality that do not require external data; most of these focus on the measurement of noise in the data. |
|  |  |  | Relevance | N/A |  |
|  |  |  | Comparability | N/A |  |

| Prang *et al.* 2022 [27] | To identify the key characteristics that a clinical registry must possess to be considered of high quality and identify which characteristics are essential to support the conduct of a registry-based randomized controlled trial (RRCT). | Clinical registry | Accuracy | The extent to which the registered data are in conformity with the truth. | This review summarizes the considerable work undertaken to determine the criteria with which to judge the suitability of a clinical registry to support a RRCT. Registries that have been specifically designed to support a RRCT should be adequately resourced to ensure the ongoing quality and integrity of their data and can generate high-quality clinical evidence required to satisfy regulatory requirements. |
| --- | --- | --- | --- | --- | --- |
|  |  |  | Completeness | The extent to which all necessary data have been registered. |  |
|  |  |  | Capture | The extent to which all necessary cases that could have been registered have actually been registered. |  |
|  |  |  | Timeliness | The rapidity with which a registry can collect, process, and report sufficiently reliable and complete data. |  |
| Ozonze *et al.* 2023 [29] | To identify necessary features and considerations that could guide EHR data quality assessment tooling, not limited to dimensions and assessment methods. | Electronic health record | Conformance | Focuses on data quality features that describe the compliance of the representation of data against internal or external formatting, relational, or computational definitions. | This review shows that automating EHR data quality assessment is gaining traction. However, there appears to be a general lack of clarity surrounding the processes brought about by the contextual nature of data quality requirements, heterogeneity of EHR data, and the challenge of developing measurements for inspecting datasets. |
|  |  |  | *Value* | This seeks to determine of recorded data elements are in agreement with a prespecified, constraint – driven data architecture. |  |
|  |  |  | *Relational* | This seeks to determine if recorded data elements are in agreement with additional structural constraints imposed by the physical database structure that store data values. |  |
|  |  |  | *Computational* | This seeks to determine if computations used to create derived values from existing variables yield the intended results either within a data set or between data sets, when programs are based on identical specifications. |  |
|  |  |  | Completeness | Focuses on features that describe the frequencies of data attributes present in a data set without reference to data values. |  |
|  |  |  | Plausibility | Focuses on features that describes the believability or truthfulness of data values. |  |
|  |  |  | *Unique* | This seeks to determine of objects appear multiple times in settings where they should not be duplicated or cannot be distinguished within a database or when compared with an external reference. |  |
|  |  |  | *Temporal* | This seeks to determine if observed data values, distributions, or densities agree with local or common knowledge or from comparisons with external sources that are deemed to be trusted or relative gold standards |  |
|  |  |  | *Atemporal* | This seeks to determine of time-varying variables change values as expected based on known temporal properties or across 1 or more external comparators or gold standards. |  |

| Syed *et al.* 2023 [23] | To develop a consolidated digital health data quality dimension and outcome (DQ-DO) framework to provide insights into 3 research questions:   - What are the dimensions of digital health DQ? - How are the dimensions of digital health DQ related? - What are the impacts of digital health DQ? | N/A | Accuracy | The degree to which data reveal the truth about the event being described. | The DQ-DO framework provides health care executives with insights into DQ issues and which digital health-related outcomes they have an impact on. The review identified four core implications to motivate future research:   - Pay equal consideration to all dimensions of DQ. - Seek to empirically assess the DQ-DO framework using a mixed methods case study design. - Identify the root causes of the digital health DQ issues. - Develop interventions for mitigating DQ issues or preventing them from arising. |
| --- | --- | --- | --- | --- | --- |
|  |  |  | Consistency | Absence of differences between data items representing the same objects based on specific information requirements. |  |
|  |  |  | Completeness | The absence of data at a single moment over time or when measured at multiple moments over time. |  |
|  |  |  | Contextual validity | Assessment of DQ is dependent on the task at hand. |  |
|  |  |  | Accessibility | How feasible it is for users to extract the data of interest. |  |
|  |  |  | Currency | The degree to which data represent reality from the required point in time. |  |
