# Supplementary material for: Frameworks, Dimensions, Definitions of Aspects, and Assessment Methods for the Appraisal of Quality of Health Data for Secondary Use: Comprehensive Overview of Reviews
Source: JMIR Med Inform. 2024 Mar 6;12:e51560. doi: 10.2196/51560 (PMC10955383; doi:10.2196/51560)

Figure S1. The frequency of all dimensions with definitions in each review.


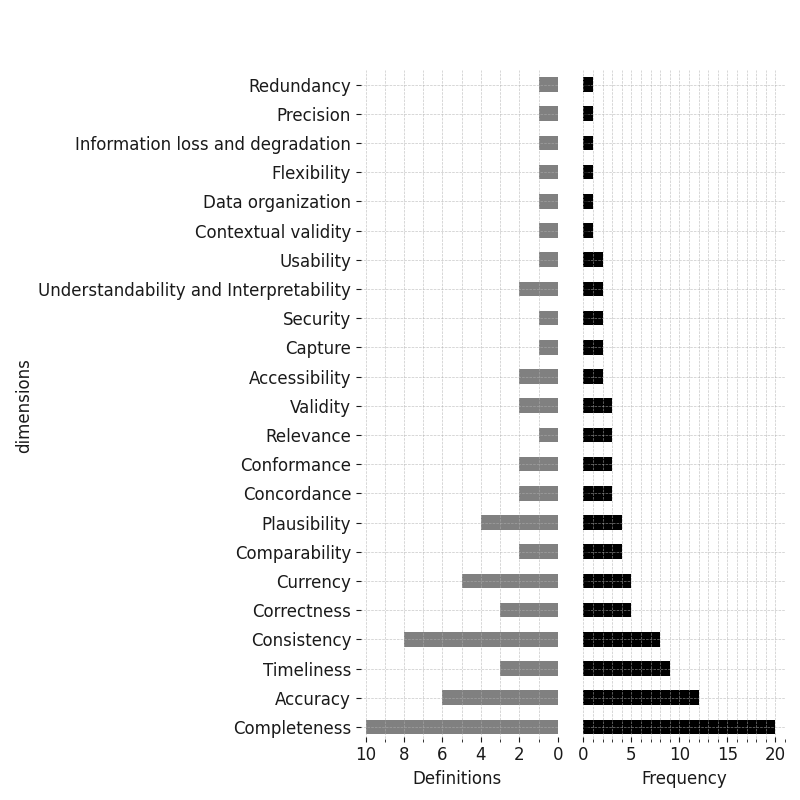


Figure S2. Assessment methods per dimension.


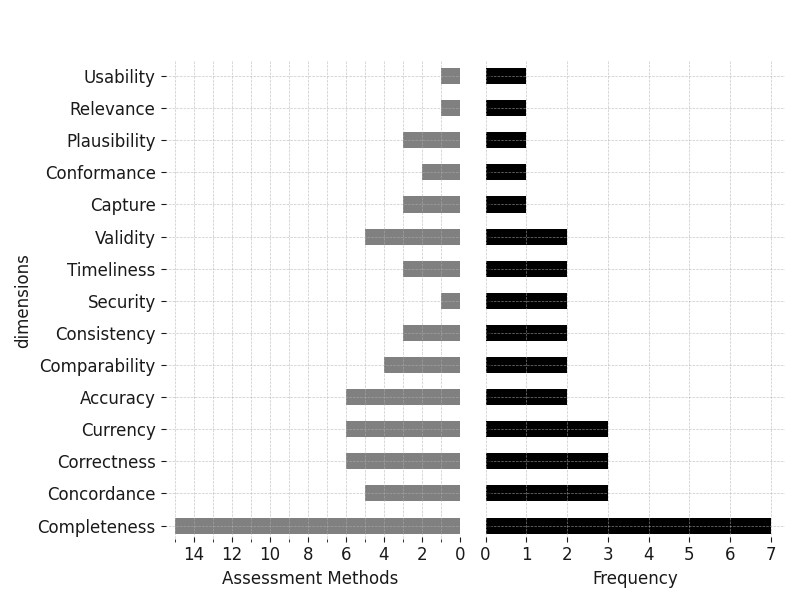

Supplement: Multimedia Appendix 3 [file medinform_v12i1e51560_app3.docx]
